# Potential Inhibitors Targeting Papain-Like Protease of SARS-CoV-2: Two Birds With One Stone

**DOI:** 10.3389/fchem.2022.822785

**Published:** 2022-02-23

**Authors:** Haihai Jiang, Peiyao Yang, Jin Zhang

**Affiliations:** ^1^ School of Basic Medical Sciences, Nanchang University, Nanchang, China; ^2^ Queen Mary School, Nanchang University, Nanchang, China

**Keywords:** COVID-19, SARS-CoV-2, papain-like protease, inhibitor, crystal structure

## Abstract

Severe acute respiratory syndrome Coronavirus-2 (SARS-CoV-2), the pathogen of the Coronavirus disease-19 (COVID-19), is still devastating the world causing significant chaos to the international community and posing a significant threat to global health. Since the first outbreak in late 2019, several lines of intervention have been developed to prevent the spread of this virus. Nowadays, some vaccines have been approved and extensively administered. However, the fact that SARS-CoV-2 rapidly mutates makes the efficacy and safety of this approach constantly under debate. Therefore, antivirals are still needed to combat the infection of SARS-CoV-2. Papain-like protease (PLpro) of SARS-CoV-2 supports viral reproduction and suppresses the innate immune response of the host, which makes PLpro an attractive pharmaceutical target. Inhibition of PLpro could not only prevent viral replication but also restore the antiviral immunity of the host, resulting in the speedy recovery of the patient. In this review, we describe structural and functional features on PLpro of SARS-CoV-2 and the latest development in searching for PLpro inhibitors. Currently available inhibitors targeting PLpro as well as their structural basis are also summarized.

## Introduction

Severe acute respiratory syndrome coronavirus 2 (SARS-CoV-2), causing the coronavirus disease 2019 (COVID-19) (Wu F. et al., 2020; Zhu et al., 2020), is the third highly pathogenic human coronavirus in this century after the SARS-CoV emerged in 2003 (Stadler et al., 2003) and the Middle East Respiratory Syndrome Coronavirus (MERS-CoV) emerged in 2012 (Chafekar and Fielding, 2018). Although it has a relatively lower mortality rate, SARS-CoV-2 exhibits a higher transmission efficiency compared to SARS-CoV and MERS-CoV (Madewell et al., 2020; Patel et al., 2020). The rapid spread of SARS-CoV-2 has continued to cause a worldwide pandemic and posed a serious threat to global public health since the beginning of 2020. The SARS-CoV-2 infection mostly affects the lungs and causes symptoms of varying degrees of morbidity, ranging from asymptomatic infection to mild infection with flu-like illness or severe infection with lung injury (Chen et al., 2020; Huang et al., 2020; Wang et al., 2020). COVID-19 outbreak has resulted in over 320 million confirmed cases as of 16 January 2022, including nearly 5.5 million deaths (https://www.who.int/publications/m/item/weekly-epidemiological-update-on-covid-19---18-january-2022). Though vaccines have been administered on a large scale, this viral disease is far from being controlled, in particular, due to the occurrence of cumulative mutations. Given the ongoing pandemic and disruptive impact, there is still an urgent need to develop new antiviral strategies for the prompt and effective therapy of SARS-CoV-2 infection.

SARS-CoV-2 is an enveloped, single-stranded, and positive-sense RNA virus (+ssRNA) which belongs to the *Coronaviridae* family (Khailany et al., 2020; Zhou et al., 2020). Coronaviruses can be divided into four groups indicated with the Greek letters α, β, γ, and δ, respectively (Su et al., 2016). Along with the newly emerged SARS-CoV-2, seven coronaviruses are currently able to infect humans, among which HCoV-NL63 and HCoV-229E belong to α coronavirus, while the others (HCoV-HKU1, HCoV-OC43, SARS-CoV, MERS-CoV, and SARS-CoV-2) belong to β coronavirus (Ye et al., 2020). Similar to other human coronaviruses, the genome of SARS-CoV-2 has two open reading frames that encode two replicase polyproteins, namely pp1a and pp1ab. The polyproteins are digested into sixteen mature non-structural proteins (nsp1-16) by two cysteine proteases, chymotrypsin-like protease (3CLpro or Mpro) encoded by nsp5 and papain-like protease (PLpro) encoded by nsp3. Mpro cleavage results in the releasing of the functional nsp4-16, while PLpro cleavage results in the maturation of nsp1-3 (Fehr and Perlman, 2015).

Importantly, SARS-CoV-2 PLpro possesses additional function of inhibiting interferon related antiviral responses of the host (Klemm et al., 2020; Shin et al., 2020). Given that SARS-CoV-2 causes a substantially higher mortality rate in elderly patients with compromised immune systems (Ruan 2020; Wu and McGoogan 2020), viral factors that mitigate or evade from immune responses are desirable drug targets. Thus, therapy by targeting SARS-CoV-2 PLpro can not only suppress viral infection but also promote antiviral immunity, which is similar to killing two birds with one stone. Herein, this review updates the recent research progress in the structure and function of SARS-CoV-2 PLpro and the discovery of PLpro inhibitors against COVID-19.

## Structural and Functional Features of SARS-CoV-2 PLpro

**FIGURE 1:**
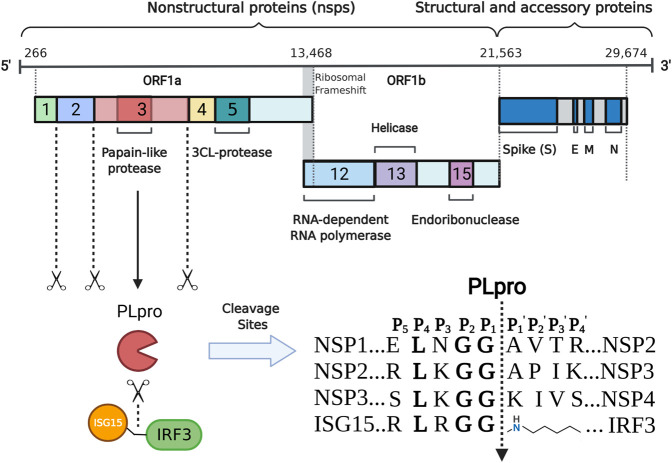
Schematic representation of the production and function of SARS- CoV- 2 PLpro. The positive- strand RNA (numbers indicate nucleotide position) including the ORF 1a/b, structural proteins S (spike), E (envelope), M (membrane) and N (nucleocapsid) are shown. PLpro is encoded by part of nsp3. It is able to mature nsp1- 3 and preferentially cleave the ubiquitin- like protein ISG15 from IRF3 by recognizing specific cleavage sites.

The genome of SARS-CoV-2 is approximately 30 kb in size and contains at least 12 open reading frames (ORF) that flanked by 5′-cap and 3′-poly(A) tail (Wu A. et al., 2020; Zhang RH. et al., 2020). The 3′-terminal one-thirds of the SARS-CoV-2 genome encodes four structural proteins (spike protein, envelope protein, membrane protein, and nucleocapsid protein) and several accessory proteins, while two-thirds of the SARS-CoV-2 genome at the 5′-terminal encodes two replicase polyproteins, namely pp1a (about 450 KD) and pp1ab (about 750 KD) (Figure 1). These two polyproteins can be cleaved by virus-encoded PLpro and 3C-like protease into 16 non-structural proteins. Many of them are localized to the double membrane like vesicles and assemble into a replication complex on the cytoplasmic face of the endoplasmic reticulum (Knoops et al., 2008; Klein et al., 2020). PLpro is part of the largest non-structural protein nsp3 and is highly conserved (Lei et al., 2018). Often two copies, referred to as PL1pro and PL2pro, are found in coronaviruses and the two PLpros show distinct substrate specificity in different coronaviruses (Woo et al., 2010; Mielech et al., 2014). For example, PL1pro of mouse hepatitis virus processes between nsp1/2 and between nsp2/3, while PL2pro processes between nsp3/4 (Bonilla et al., 1997; Kanjanahaluethai and Baker, 2000). In the case of HCoV-NL63, PL1pro cleaves between nsp1/2, while PL2pro cleaves between nsp2/3 and between nsp3/4 (Chen et al., 2007). However, like SARS-CoV and MERS-CoV, only one functional PLpro is encoded in SARS-CoV-2. After being released from nsp3 through autocleavage, SARS-CoV-2 PLpro recognizes the common motif LXGG(A/K)X between nsp1/2, nsp2/3, and nsp3/4 (X represents any type of amino acid) and cleaves between glycine and alanine/lysine residues (Anirudhan et al., 2021), which is essential for coronavirus RNA synthesis and viral survival (Shamsi et al., 2021; Yadav et al., 2021). Enzyme activity analysis revealed that the S2 site of PLpro strictly recognizes glycine and the S4 site preferentially recognizes amino acids with hydrophobic side chains, while the S3 site has broader substrate specificity and can recognize any type of amino acid (Rut et al., 2020).

Beyond the role in cleaving the viral polypeptide, PLpro also participates in regulating host antiviral innate immunity through antagonising ubiquitin and ubiquitin-like modifications (Shin et al., 2020; Klemm et al., 2020). The host elicits various defense strategies to thwart viral infection. During viral invasion, the host could recognize the specific viral components through pattern recognition receptors and subsequently produce type I interferon and proinflammatory cytokines to establish the first line of host defense against viral infection. Ubiquitins and ubiquitin- like protein ISG15, an interferon- induced protein, are important post- translation modifying processes and have emerged as crucial players at this stage (Zheng and Gao, 2019, Zheng and Gao, 2020; Perng and Lenschow, 2018; McClain and Vabret, 2020). In response, virus often repurposes its protease to evade host antiviral immunity through deubiquitinating and deISGylating activities (James et al., 2011; Swatek et al., 2018). In the case of SARS-CoV-2, PLpro can remove ubiquitin and ISG15 modifications from host proteins by cleaving the consensus site (LXGG) (Figure 1) (Swaim et al., 2020). Similar to SARS-CoV PLpro, SARS-CoV-2 PLpro shows detectable activity for K48-linked ubiquitin chains but not K63, even though at a substantially slower rate (Freitas et al., 2020; Rut et al., 2020; Shin et al., 2020). Unlike SARS-CoV PLpro that predominantly cleaves K48 ubiquitin chains, PLpro of SARS-CoV-2 shows an enhanced deISGylation activity (Freitas et al., 2020; Rut et al., 2020; Shin et al., 2020). However, the deISGylation activity is sensitive to species-species differences (Freitas et al., 2020). SARS-CoV-2 PLpro appears to prefer ISG15s from sheep and the vesper bat, but shows no protease activity for the ISG15 substrate from fish. The deISGylase activities are moderate for ISG15s from human, pig, camel, and mouse, while weak for ISG15s from Egyptian fruit bat, hedgehog, and northern tree shrew. The preference of PLpro in processing ISG15 substrates may indicate the species that SARS-CoV-2 can productively infect.

**FIGURE 2:**
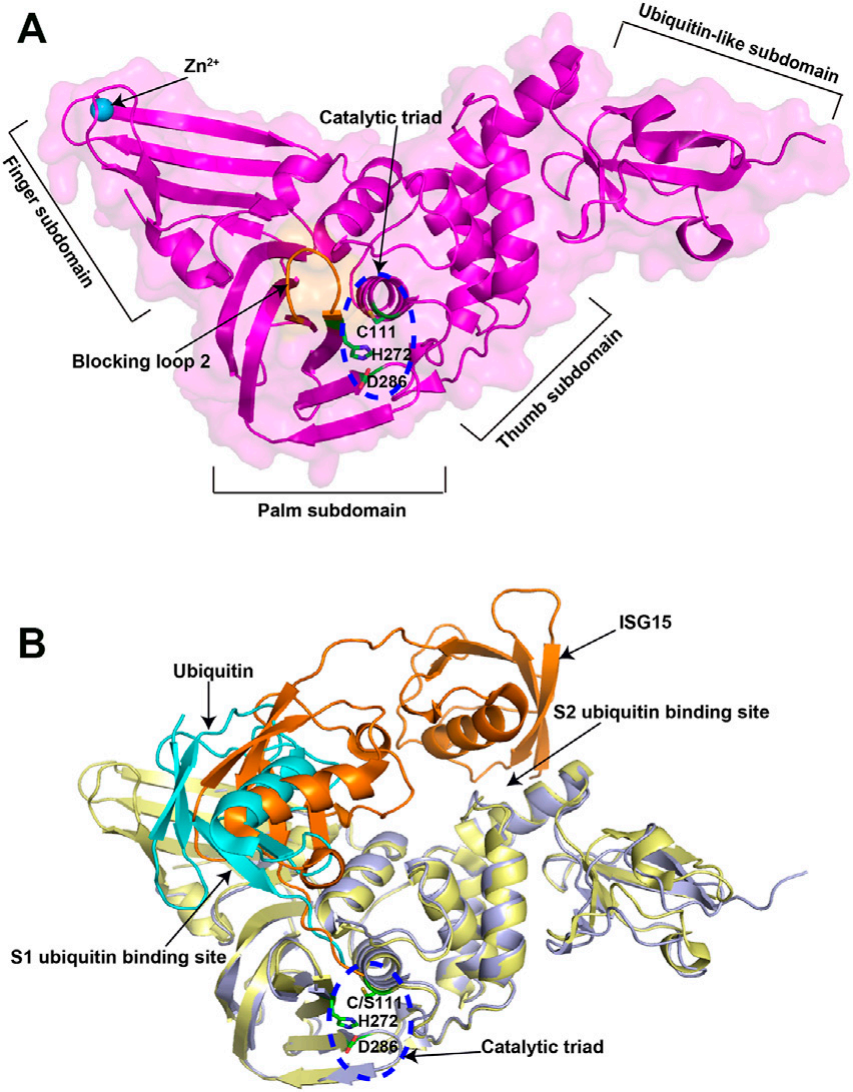
Crystal structures of SARS-CoV-2 PLpro in apo and substrate-bound forms. **(A)** Crystal structure of SARS-CoV-2 PLpro (PDB ID: 6WZU). The four distinct subdomains are indicated. The catalytic triad is shown in the blue dashed circle. **(B)** Superposition of ubiquitin/PLpro complex (PDB ID: 6XAA) with mouse-ISG15/PLpro complex (PDB ID: 6YVA). The catalytic triad is shown in the blue dashed circle. The two ubiquitin binding sites are indicated. The ubiquitin is colored in cyan, while the mouse ISG15 is colored in orange.

Recently, the crystal structure of the 315-residue SARS-CoV-2 PLpro has been solved (Osipiuk et al., 2021). Similar to PLpros of SARS-CoV and MERS-CoV PLpro, SARS-CoV-2 PLpro contains a ubiquitin-like domain at N-terminal and a catalytic domain at C-terminal, which can be divided into three subdomains, namely thumb, palm and finger subdomains (Figure 2A). The catalytic site of SARS-CoV-2 PLpro contains a classical catalytic triad, consisting of Cys111, His273, and Asp287, and is situated at the interface between thumb and palm subdomains. A 6-amino-acid (267–272 residues) flexible loop named blocking loop 2 (BL2) near the catalytic site plays an important role in controlling the access to the active site (Ratia et al., 2008). The finger subdomain contains a zinc-ribbon region and zinc ion binding is necessary for enzyme catalysis and structural integrity of PLpro (Tian et al., 2021; Zhao et al., 2021).

PLpro possesses two ubiquitin binding sites (S1 and S2) for the recognition of its substrates, ubiquitin and ISG15, with the structural basis of which has also been elucidated (Klemm et al., 2020; Shin et al., 2020). S1 ubiquitin binding site recognizes the C-terminal domain of ISG15 through a different binding orientation compared with ubiquitin (Figure 2B). S2 ubiquitin binding site not only recognizes the N-terminal domain of ISG15, but also provides exquisite specificity for the distal ubiquitin of K48-linked diubiquitin chains. Residue Phe69 and Thr75 in the S2 ubiquitin-binding site are key amino acids for PLpro to interact with Ile44 in ISG15, which may contribute to the enhanced affinity for ISG15. However, these observations are based on the available structures of SARS-CoV-2 PLpro in complex with ubiquitin or mouse full-length ISG15. It would be of interest in future to determine the structure of human full-length ISG15 or K48-diubiquitin in complex with SARS-CoV-2 PLpro.

Nevertheless, these data reveal the 3D structures of PLpro at both apo state and substrate-binding state, which facilitates the PLpro based drug design. Molecules that mimic the hydrophobic interaction with SARS-CoV-2 PLpro observed in its substrates or alter the conformation of BL2 in the active site will affect the protease activity.

## Strategies in PLpro Inhibitor Development

### Drug Repurposing

Drug repurposing refers to the discovery of new therapeutic uses of clinically available drugs. It is a prompt strategy in searching of medications for COVID-19 therapy since the traditional drug discovery process can be costly and takes decades to complete (Aherfi et al., 2021; Hijikata et al., 2021; Mslati et al., 2021; Shende et al., 2021). As repurposed drugs have gone through clinical trial, important parameters of these drugs, such as inhibitory potential, cell permeability, bio-availability, and safety, have been well characterized and can be available in a short time in face of pandemics (Pushpakom et al., 2019). Since the outbreak of COVID-19 in late December, 2019, drug repurposing has been employed to deal with this worldwide health emergency by targeting PLpro, even though such efforts were not as successful as hoped at the beginning of the pandemic.

The disclosure of high resolution crystal structures of SARS-CoV-2 PLpro has made virtual screening a useful strategy in drug repurposing (Gao et al., 2021; Ma et al., 2021; Osipiuk et al., 2021). In several studies, the crystal structure of SARS-CoV-2 PLpro was included in a molecular docking analysis to identify PLpro inhibitors (Choudhury et al., 2021; Hosseini et al., 2021). In addition, pharmacophore based screen is an alternative method to obtain potential inhibitors (Kouznetsova et al., 2020). Although virtual screening is a useful strategy for drug repurposing, further *in vitro* and *in vivo* tests are necessary to confirm its activities against SARS-CoV-2 PLpro.

High-throughput screening (HTS) is another useful strategy in drug repurposing. It selects the most promising candidates from a large number of molecules via experimental approaches. Smith et al. developed a cell-based luciferase complementation assay to evaluate the inhibition of known drugs against SARS-CoV-2 viral PLpro (Smith et al., 2020). Based on the fluorescence-based enzymatic inhibition assay, several groups have identified potential inhibitors of SARS-CoV-2 PLpro with the IC_50_ value under 10 μM (Cho et al., 2021; Xu et al., 2021; Zhao et al., 2021). Although not all the identified compounds exhibited good performance in a cell-based assay, they could serve as a starting point for further chemical modification.

### Discovery of New Drug Leads

Discovery of new drug leads is an important step to develop a novel drug. It involves the employment of a wide range of technologies including virtual screening, high-throughput screening and structure-based drug design (Guido et al., 2011). These interdisciplinary expertises are complementary and comprehensive application of a variety of techniques in identification of new lead compounds is feasible. The general process of leads identification includes two essential steps, namely hits generation and subsequent hits validation. Compared with drug repurposing, screenings for hit compounds, either *in vitro* or *in silico*, incorporate a much larger scale of molecules including microbial metabolites, natural products and marine-derived bio-active compounds (Cragg and Newman, 2013; Kumar et al., 2021; Quimque et al., 2021). Since the outbreak of COVID-19, the widespread use of combinatorial screening has revealed a large mount of hit compounds that specifically target SARS-CoV-2 PLpro (Amin et al., 2021; Gogoi et al., 2021; Goyzueta-Mamani et al., 2021; Hajbabaie et al., 2021; Jade et al., 2021; Jamalan et al., 2021; Kumar et al., 2021; Li et al., 2021; Quimque et al., 2021; Rahul and Sarkar, 2021; Rudrapal et al., 2021; Stasiulewicz et al., 2021). Hits generated through the initial screens are further validated with an aim to choose the best ones to serve as leads for drug developments. As not all the identified lead compounds exhibited desired activities, co-crystallization of these effective inhibitors with PLpro is often adopted to investigate how the two molecules interact (Wang et al., 2021), which can inform further lead optimization and future drug design.

### Optimization of Lead Compounds

Once a potential lead is identified, lead optimization is pressing as the potency, selectivity, or pharmacokinetic parameters of leads identified may not be satisfactory. Thus, optimization of lead compounds is a crucial step in the development of new drugs. As an example of success, Shan et al. optimized GRL0617 based on the GRL0617/PLpro complex structure and found analogue **19** has favorable potency and selectivity against SARS-CoV-2 PLpro (Shan et al., 2021). Also, Ma et al. optimized the Jun9-13-7 and Jun9-13-9 (Ma et al., 2021), while Weglarz-Tomczak et al. modified ebselen (Weglarz-Tomczak et al., 2021). Both optimized inhibitors are more potent than parent compounds. It is worth noting that all these optimized inhibitors suppress the PLpro activity at nanomolar level, representing the most potent inhibitors at the time of this manuscript submission.

## Potential SARS-CoV-2 PLpro Inhibitors

Many inhibitors have been identified against SARS-CoV-2 PLpro by using the strategies as stated above, even though no PLpro inhibitor has been approved by the US Food and Drug Administration (FDA) for marketing. Cysteine protease inhibitors can be divided into two categories, namely, covalent and non-covalent inhibitors. Covalent inhibitors form a C-S thioether linkage with the catalytic residue cysteine, while the non-covalent inhibitors interact with the protease through non-covalent binding which is always a reversible process (Aljoundi et al., 2020). SARS-CoV-2 PLpro inhibitors identified to date are mostly non-covalent, including naphthalene-based inhibitors, FDA approved drugs and natural products. As follows, we review the promising inhibitors currently known that target SARS-CoV-2 PLpro with the representative ones summarized in Table 1.

**TABLE 1:** Representative potential inhibitors of SARS-CoV-2 PLpro.

Inhibitor	Enzyme inhibition activity (IC_50_)	Antiviral potency (EC_50_)	Crystal structure in complex with PLpro (PDB ID)	Molecular structure	References
VIR250	NA	NA	6WUU	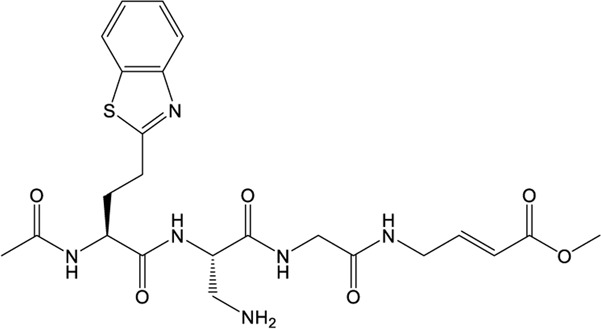	Rut et al. (2020)
VIR251	NA	NA	6WX4	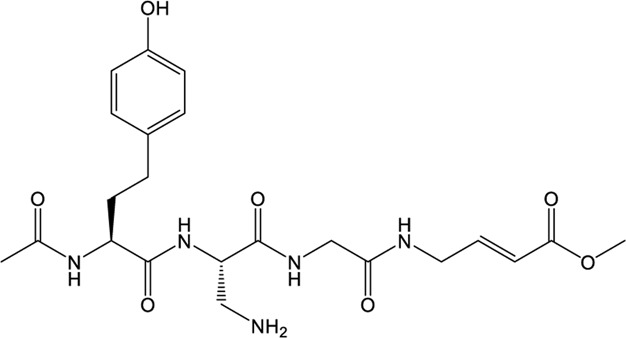	Rut et al. (2020)
GRL0617	2.05 ± 0.12 μM	Vero E6: 23.64 ± 4.72 μM; Caco2-hACE2: 19.96 ± 8.82 μM	7CMD	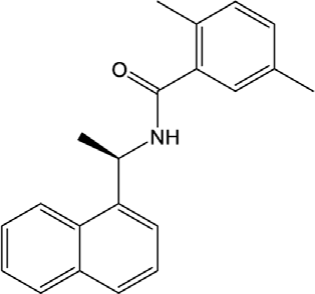	Ma et al. (2021); Gao et al. (2021)
XR8-24	0.56 ± 0.03 μM	A549: 1.4 ± 0.1 μM	7LBS	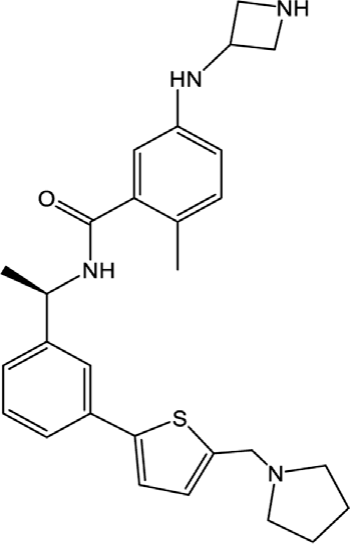	Shen et al. (2021)
XR8-23	0.39 ± 0.05 μM	A549: 1.2 ± 0.2 μM	NA	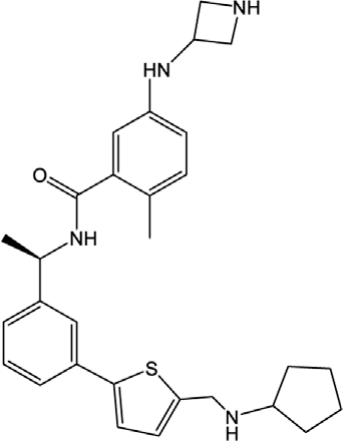	Shen et al. (2021)
19	0.44 ± 0.05μM	hACE2-HeLa: 0.18 ± 0.10 μM	NA	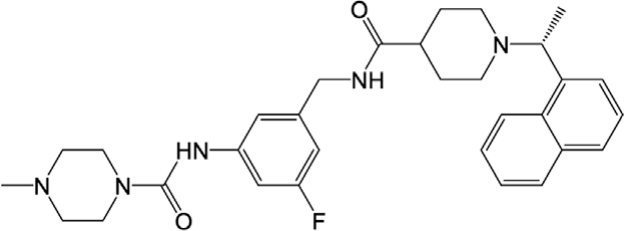	Shan et al. (2021)
Jun9-72–2	0.67 ± 0.08 μM	Vero E6: 6.62 ± 0.31 μM; Caco2-hACE2: 7.90 ± 2.40 μM	7SDR	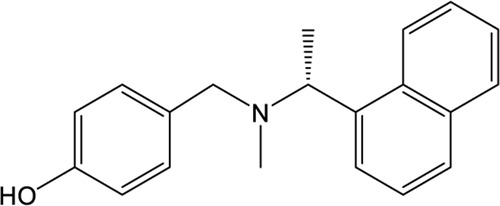	Ma et al. (2021)
Jun9-75–4	0.62 ± 0.06 μM	Vero E6: 7.88 ± 1.44 μM; Caco2-hACE2: 12.48 ± 3.43 μM	NA	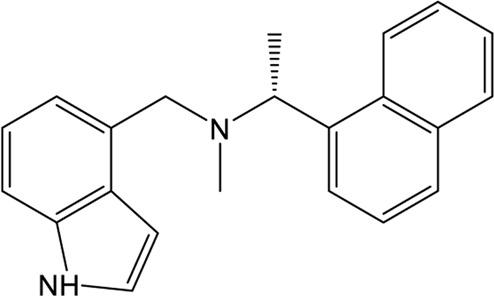	Ma et al. (2021)
Cryptotanshinone	5.63 ± 1.45 μM	Vero E6: 0.70 ± 0.09 μM	NA	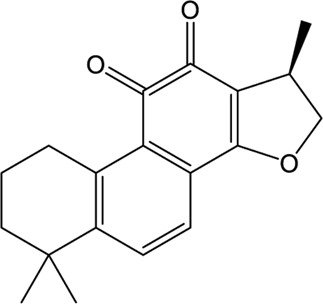	Zhao et al. (2021)
Tanshinone l	2.21 ± 0.10 μM	Vero E6: 2.26 ± 0.11 μM	NA	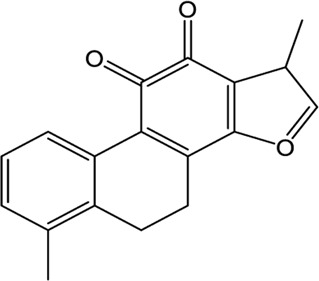	Zhao et al. (2021)
Dihydrotanshinone I	0.5861 μM	Vero E6: 8.148 µM	NA	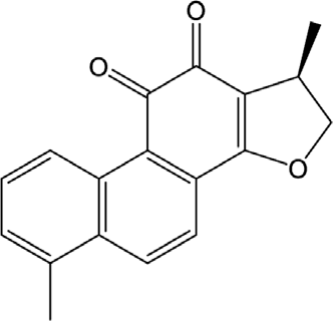	Lim et al. (2021)
YM155	2.47 ± 0.46 μM	Vero E6: 0.17 ± 0.02 μM	7D7L	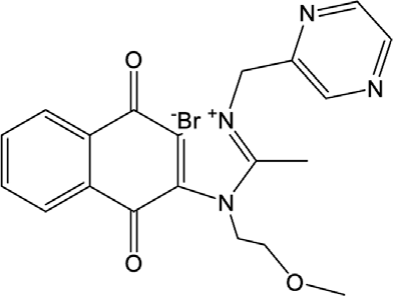	Zhao et al. (2021)
Ebselen	2.02 ± 1.02 μM	Vero E6: 4.67 ± 0.80 µM	NA	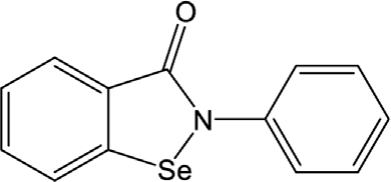	Jin et al. (2020); Ma et al. (2020)
Rac5c	0.81 μM	NA	NA	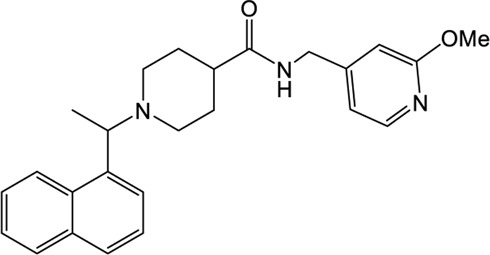	Klemm et al. (2020)
1d	0.236 μM	NA	NA	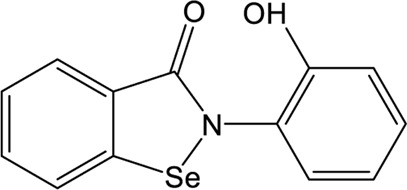	Weglarz-Tomczak et al. (2021)
1e	0.256 μM	NA	NA	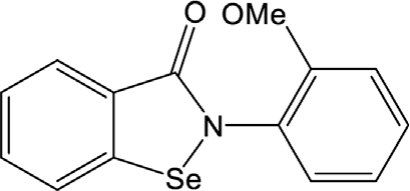	Weglarz-Tomczak et al. (2021)
Disulfiram	7.52 ± 2.13 μM	NA	NA	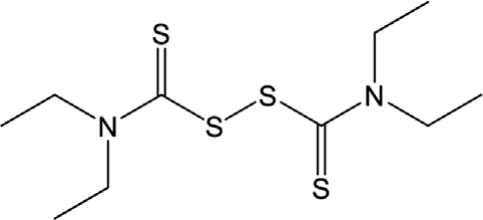	Sargsyan et al. (2020)
6-TG	NA	Vero E6: 2.13 ± 1.16 µM	NA	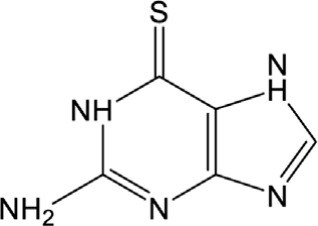	Bayoumy et al. (2020)

NA: Not available.

### Naphthalene-Based Inhibitors

**FIGURE 3:**
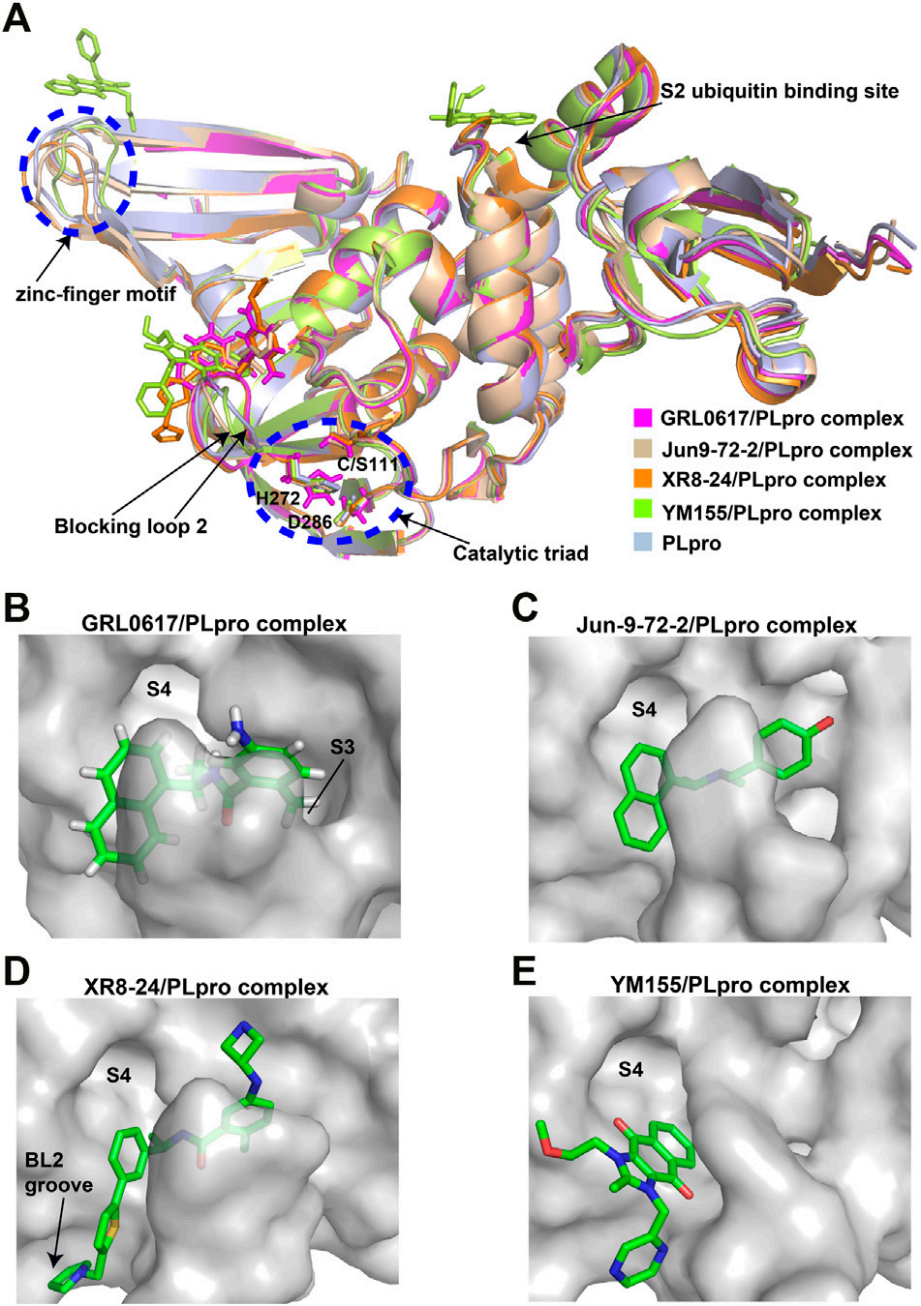
Crystal structure of SARS-CoV-2 PLpro in complex with non-covalent inhibitors. **(A)** Superposition of PLpro (light blue; PDB ID: 6WZU) with GRL0617/PLpro (magentas; PDB ID: 7CMD), Jun9-72-2/PLpro (wheat; PDB ID: 7SDR), XR8-24/PLpro (orange; PDB ID: 7LBS), and YM155/PLpro (limon; PDB ID: 7D7L) complexes. The catalytic triad and the zinc-finger motif are shown in the blue dashed circle. The blocking loop 2 and the S2 ubiquitin binding site are indicated. **(B)** Surface presentation of GRL0617 binding pocket. PLpro is shown as gray surface, while GR0617 is shown as sticks. S3 and S4 pockets are labeled. **(C)** Surface presentation of Jun9-72-2 binding pocket. PLpro is shown as gray surface, while Jun9-72-2 is shown as sticks. S4 pocket is labeled. **(D)** Surface presentation of XR8-24 binding pocket. PLpro is shown as gray surface, while XR8-24 is shown as sticks. S4 pocket and BL2 groove are labeled. **(E)** Surface presentation of YM155 binding pocket. PLpro is shown as gray surface, while YM155 is shown as sticks. S4 pocket is labeled.

GRL0617, a naphthalene-based drug, was previously developed as a non-covalent inhibitor of SARS-CoV PLpro (Ratia et al., 2008). In various high-throughput screen studies for SARS-CoV-2 PLpro inhibitors, it has stood out for its potent protease inhibition and antiviral activities, with the IC_50_ being around 2.0 μM and EC_50_ being around 20 μM, respectively (Table 1) (Gao et al., 2021; Fu et al., 2021; Osipiuk et al., 2021; Klemm et al., 2020; Freitas et al., 2020). Several groups then resolved the crystal structure of SARS-CoV-2 PLpro in complex with GRL0617 and revealed its inhibitory mechanism (Gao et al., 2021; Fu et al., 2021; Osipiuk et al., 2021; Ma et al., 2021). According to the structural information, GRL0617 fits well with the substrate cleft with its aromatic ring and naphthalene group inserted into S3 and S4 pockets, respectively (Figures 3A,B). Unlike other inhibitors such as VIR251 (described below), which stabilizes the BL2 in an open conformation, GRL0617 keeps this loop in a close conformation (Figure 3A) (Rut et al., 2020; Gao et al., 2021). Thus, GRL0617 prevents the substrate from entering the active site and is therefore a competitive inhibitor. These findings inspire the discovery of new generation of naphthalene based PLpro inhibitors.

Osipiuk et al. reported several GRL0617 analogues that also show good inhibition of SARS-CoV-2 PLpro (Osipiuk et al., 2021). However, none of them show more potent inhibition of SARS-CoV-2 PLpro compared with GRL0617. Later, Ma et al. reported two lead compounds, Jun9-13-7 and Jun9-13-9, that exhibits similar potency compared with GRL0617 (Ma et al., 2021). Encouragingly, two of the optimized forms, Jun9-72-2 and Jun9-75-4, show more potent enzymatic inhibition and antiviral activity compared to GRL0617. The IC_50_ and EC_50_ values of Jun9-72-2 and Jun9-54-7 are several-fold lower than those of GRL0617 (Table 1), thus making them very effective PLpro inhibitors. Jun9-72-2 adopts a similar binding model with GRL0617 (Figures 3A,C). Shan et al. focused on the structure-based optimization of GRL0617 for searching for improved GRL0617 analogues (Shan et al., 2021), as GRL0617 lacks sufficient potency for development as an antiviral agent. Finally, 9 GRL0617 analogues carrying a shared naphthyl subunit were found to be more potent than GRL0617. Among these, analogue **19** exhibits an inhibitory activity against SARS-CoV-2 PLpro with the IC_50_ value of 0.44 μM and an antiviral activity against SARS-CoV-2 with the IC_50_ value of 0.18 μM (Table 1). Moreover, analogue **19** shows no significant cross-inhibition against another 10 deubiquitinating enzymes (DUB) or DUB-like proteases even at the concentration of 10 μM (Shan et al., 2021). Collectively, analogue **19** is a promising SARS-CoV-2 PLpro inhibitor in virtue of its high degree of potency and selectivity.

In addition to GRL0617, another three previously identified naphthalene-based inhibitors, namely, rac3j, rac3k, and rac5c (the racemic version of 3j, 3k, and 5c, respectively), also show a promising inhibitory activity against SARS-CoV-2 PLpro. Rac5c is the best one with an *in vitro* IC_50_ value of 0.81 μM (Table 1) (Klemm et al., 2020). In the antiviral assay, rac5c could protect SARS-CoV-2 infected Vero cells from cytopathic effect without causing cell toxicity at a concentration of 11 μM, indicating the striking antiviral effects.

### XR8-23/XR8-24

Shen et al. attached more attention in optimizing the potency of GRL0617 by exploring the engagement of additional binding sites beyond those utilized by GRL0617 (Shen et al., 2021). ZN-2–184, a derivative of GRL0617 with an azetidine substitution on the phenyl ring, yielded a 2-fold improved affinity through extending interaction with Glu167 of PLpro. ZN-3-80, a derivative of ZN-2-184 with the naphthyl group replaced by a biaryl group, showed improved metabolic stability compared with GRL0617. Further attempt explored the derivatization of the 2-phenylthiophene scaffold (ZN-3-80) to exploit additional interactions with the BL2 groove, a promising site that has not yet been recognized by other PLpro inhibitors. Among the newly synthesized 2-phenylthiophene inhibitors, two well-designed robust ones, XR8-23 and XR8-24, display low nanomolar potency against SARS-CoV-2 PLpro (IC_50_ values of 0.39 and 0.56 μM, respectively) and low micromolar potency against viral infection in human lung epithelial A549 cells (EC_50_ values of 1.4 and 1.2 μM, respectively) (Table 1) (Shen et al., 2021), which improved greatly over GRL0617. Furthermore, XR8-23 and X8-24 shows satisfactory bioavailability after intraperitoneal injection in a mouse model, even though their *in vivo* antiviral activities remain to be investigated.

XR8-23 has a basic amine side chain extending from the thiophene group, which leads to dissociation rates slower and potency stronger than those of ZN-3-80 and GRL0617. XR8-24 contains a pyrrolidine ring extending from the thiophene group, which results in the formation of a putative hydrogen bond with Tyr264 and accordingly accounts for its superior potency. XR8-24 do not access the active site, but engage the BL2 groove to enforce the sealing of the active site, as proved by the crystal structure of XR8-24 in complex with SARS-CoV-2 PLpro (Figures 3A,D) (Shen et al., 2021).

### YM155

A high-throughput screen also identified a new lead YM155, a phase I clinical trial antineoplastic drug. It inhibits SARS-CoV-2 PLpro with an IC_50_ value of 2.47 μM and shows robust antiviral activity with an EC_50_ value of 0.17 μM (Table 1) (Zhao et al., 2021). The crystal structure of SARS-CoV-2 PLpro (with a C111S mutation) in complex with YM155 unravels a unique interaction mechanism, which may explain the strong inhibition achieved by YM155 against PLpro. In addition to targeting the substrate-binding pocket like GRL0617, YM155 also targets the thumb domain and the zinc finger motif (Figures 3A,E). Thus, YM155 not only blocks the entrance of substrate into the active site, but also hampers the molecular interactions between PLpro and ISG15 and affects the stability of the zinc-finger motif and enzyme activity.

### Natural Products

A wide range of natural products provide an ideal library for the screen and identification of new drug candidates targeting SARS-CoV-2 PLpro. Flavonoids are a kind of widely distributed plant secondary metabolites with more than 9,000 structures currently identified (Wang et al., 2018). As the largest group of polyphenolic compounds in higher plants, flavonoids have shown PLpro inhibitory effects and antiviral activities against SARS-CoV and MERS-CoV *in vitro* (Jo et al., 2020; Solnier and Fladerer, 2020). In the setting of SARS-CoV-2, several members of flavonoids, including coumaroyltyramine, cryptotanshinone, kaempferol, moupinamide, N-cis-feruloyltyramine, quercetin, and tanshinone IIa, were identified as SARS-CoV-2 PLpro ligands using an *in silico* docking analysis (Zhang D.-h. et al., 2020), despite the lack of *in vitro* evidence for their efficacy. These compounds might interfere with substrate entering the active sites through interacting with the region between the thumb and palm domains (Zhang D.-h. et al., 2020). Cryptotanshinone, one of the active ingredient from the Chinese herbal medicine, Salvia miltiorrhiza, was emphasized for its potential in inhibiting SARS-CoV-2 PLpro in another study (Zhao et al., 2021). *In vitro*, cryptotanshinone inhibits PLpro with an IC_50_ value of 5.63 μM and display antiviral activity with an EC_50_ value of 0.70 μM (Table 1), which is comparable to that of remdesivir (0.77 μM). Structurally similar with cryptotanshinone, tanshinone I possesses similar inhibition against SARS-CoV-2 PLpro (Diniz et al., 2021; Zhao et al., 2021). In addition, dihydrotanshinone I, another tanshinone derivative, could effectively inhibit the SARS-CoV-2 proliferation at an EC_50_ of 8 µM and is not cytotoxic even at high concentrations (Lim et al., 2021). Thus, tanshinones and its analogues have the potential to treat SARS-CoV-2 infection. Actually, “Xuebijing”, a complex traditional Chinese preparation consisting of Salvia miltiorrhiza, has been included in the Chinese clinical treatment strategy for COVID-19 and reduces multiple organ damage through anti-inflammation and improving immune function (Tong et al., 2020). Other natural compounds, such as EGCG (a Green Tea Catechin) (Chourasia et al., 2021), cyanovirin-N (Naidoo et al., 2021), several Terpene compounds (Diniz et al., 2021), and propolis derivatives (Yosri et al., 2021), were also extensively studied in molecular simulations and exhibited high binding affinities with SARS-CoV-2 PLpro. But further *in vitro* and *in vivo* studies are needed to verify their potential. Collectively, it indicates that these herbal compounds can be potential antivirals against SARS-CoV-2. Further studies on the mode of interaction between PLpro and these compounds may shed light on future drug discovery.

### Approved Drugs

Repurposing approved drugs is a potential alternative strategy to restrict SARS-CoV-2 infection. 6-Thioguanine (6-TG) is an orally-delivered anti-leukemia and immunosuppressant agent (Bayoumy et al., 2020). A recent study showed that 6-TG could inhibit viral replication of SARS-CoV-2 with an EC_50_ of 2.13 μM, which is similar to that of remdesivir and approximately 15-fold lower than that of GRL0617 (Swaim et al., 2021). Four clinically approved hepatitis C protease inhibitors (simeprevir, vaniprevir, paritaprevir, and grazoprevir) have also recently been shown to inhibit SARS-CoV-2 PLpro *in vitro* and viral replication in Vero-E6 cells (Bafna et al., 2021). The blockade of viral infection for these drugs display synergistic effects to the antiviral activity of remdesivir with the EC_50_ values ranging from 4.25 to 10.8 μM. In addition, simeprevir, vaniprevir, and grazoprevir exert dual inhibition of both Mpro and PLpro, while paritaprevir only targets PLpro (Bafna et al., 2021). Losartan is a clinically available drug used to treat several diseases including diabetic nephropathy and primary hypertension (Mulla and Siddiqui, 2021). Despite of a weak inhibitory effect on deubiquitinase or deISGylase activity of the SARS-CoV-2 PLpro, Losartan is effective to decrease viral replication and the EC_50_ value is 13.7 μM in the pre-infection treatment experiment (Nejat et al., 2021), suggesting that its anti-SARS-CoV-2 activity not purely depend on PLpro inhibition. Another approved drug, disulfiram, is an thiol reagent designed for alcohol aversion therapy by targeting hepatic aldehyde dehydrogenase (Lipsky et al., 2001; Krampe and Ehrenreich, 2010). It has been recently repurposed as an inhibitor of other cysteine-containing enzymes such as methyltransferase, urease, and cysteine protease of coronoviruses (Paranjpe et al., 2014; Diaz-Sanchez et al., 2016; Lin et al., 2018; Ma et al., 2020; Sargsyan et al., 2020), indicating broad-spectrum characteristics. According to pieces of evidences, disulfiram could doubly inhibit Mpro and PLpro of SARS-CoV-2 with the IC_50_ values of 9.35 and 7.52 μM, respectively (Sargsyan et al., 2020; Weglarz-Tomczak et al., 2021). The mechanism of action by disulfiram may involve the covalent interaction with Zn^2+^-bound or catalytic cysteine.

### VIR250/VIR251

**FIGURE 4:**
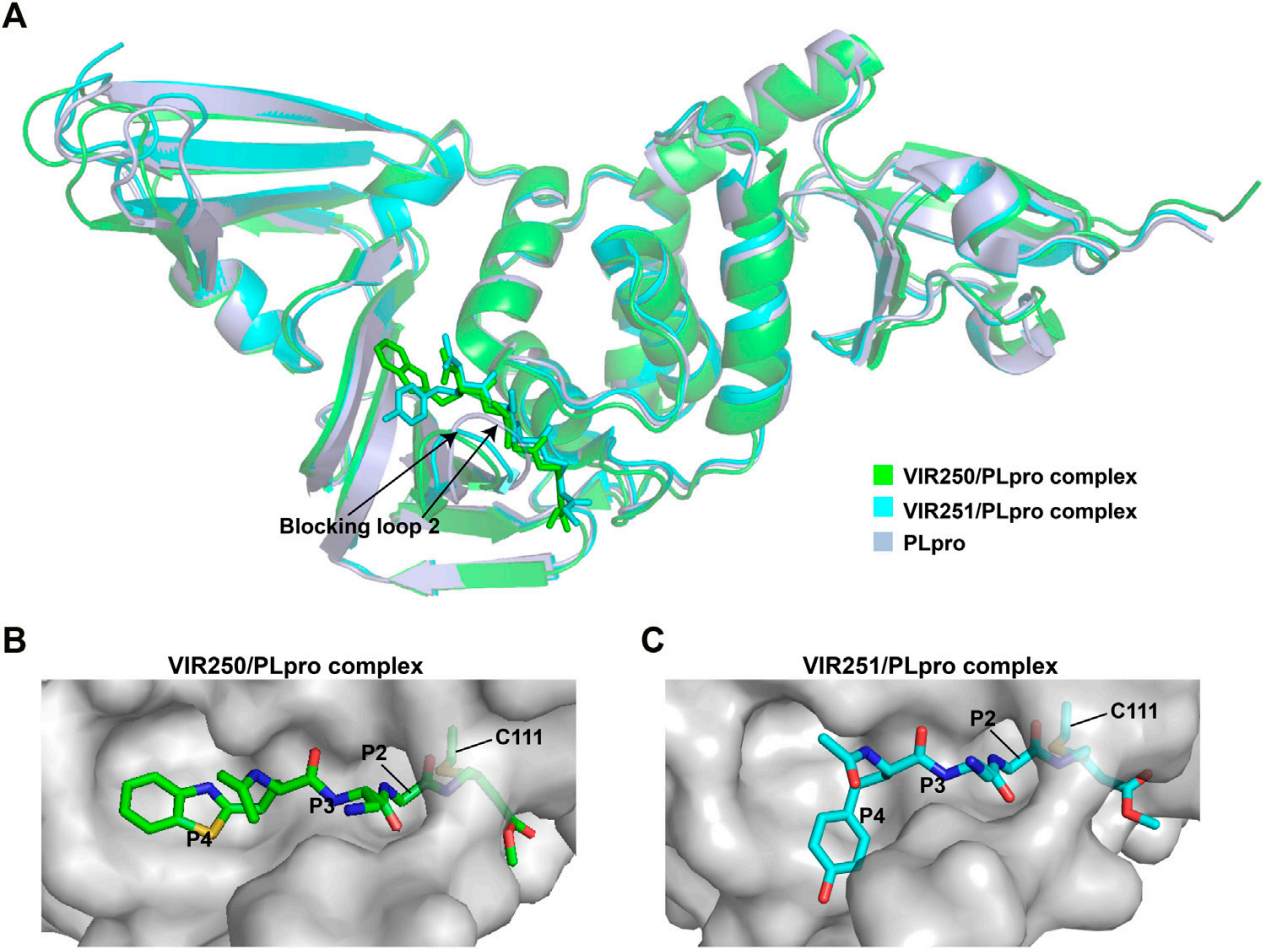
Crystal structures of SARS-CoV-2 PLpro in complex with covalent inhibitors. **(A)** Superposition of SARS-CoV-2 PLpro (light blue; PDB ID: 6WZU) with VIR250/PLpro (green; PDB ID: 6WUU) and VIR251/PLpro (cyan; PDB ID: 6WX4) complexes. The blocking loop 2 is indicated. **(B)** Surface presentation of VIR250 binding pocket. PLpro is shown as gray surface, while VIR250 is shown as sticks. The catalytic cysteine of PLpro forms a covalent bond with VIR250. The P2-P4 positions are labeled. **(C)** Surface presentation of VIR251 binding pocket. PLpro is shown as gray surface, while VIR251 is shown as sticks. The catalytic cysteine of PLpro forms a covalent bond with VIR251. The P2-P4 positions are labeled.

The peptidomimetic inhibitors VIR250 and VIR251 are the covalent inhibitors of SARS-CoV-2 PLpro identified from a combinatorial substrate library (Rut et al., 2020). These two covalent inhibitors show good inhibition against both SARS-CoV and SARS-CoV-2 PLpros with little cross-reactive to human deubiquitinating enzymes. Crystal structures of VIR250 and VIR251 in complex with PLpro were resolved to 2.79 Å and 1.65 Å, respectively (Rut et al., 2020). They have very similar molecular structures and accordingly adopt similar binding modes with PLpro (Figure 4A). Specifically, β-carbon of the glycine vinyl methyl ester (GlyVME) at the P1 position covalently links to the sulfur atom of Cys111 of SARS-CoV-2 PLpro (Figures 4B,C). In addition, P1-P3 moieties of both inhibitors engage SARS-CoV-2 PLpro mainly through polar interactions and hydrogen bonds, while P4 moiety engages PLpro through hydrophobic interactions. However, the extension of these two inhibitors at the P4 position adopts opposite orientations. Thus, additional binding space in this pocket remains to be exploited, which may inspire further drug optimization. Based on the structure of VIR251 in complex with SARS-CoV-2 PLpro, several potential inhibitors were screened against PLpro with a similar interaction mode (Delre et al., 2020; Pang et al., 2020), suggesting that VIR251, as well as VIR250, can be a good starting point for the discovery of new drugs.

### Ebselen

Ebselen is a low-molecular-weight selenoorganic drug with excellent properties in antioxidation, anti-inflammation, antiatherosclerosis, and cytoprotective effects (Azad and Tomar, 2014; Belvisi et al., 2000; Singh et al., 2017). This compound shows inhibition against Mpro and PLpro of SARS-CoV-2 with the IC_50_ values of 0.67 and 2.36 μM, respectively (Sargsyan et al., 2020; Ma et al., 2020; Jin et al., 2020; Weglarz-Tomczak et al., 2021), which are slightly more potent than disulfiram. In a cell-based assay, ebselen exhibits promising antiviral activity with the EC_50_ of  4.67 µM (Ma et al., 2020; Jin et al., 2020). The inhibition of ebselen against PLpro is an irreversible process that seems to associate with the covalent interaction between selenium atom from ebselen and sulphur atom from catalytic cysteine (Sargsyan et al., 2020; Weglarz-Tomczak et al., 2021). Subsequent modification of ebselen results in significantly increased inhibitory potency against PLpro from SARS-CoV-2. Four ebselen derivatives, namely **1d**, **1e**, **2d**, and **2e**, have IC_50_ constants in the nanomolar range, which is one order of magnitude lower than that of ebselen. Among these, **1d** and **1e** appear to be the most potent derivatives with the IC_50_ values of 236 and 256 nM, respectively (Table 1). The hydroxy (**1d**) or methoxy group (**1e**) substitution in the ortho position of the phenyl ring of ebselen may adopt additional interactions with the conserved residues in the active site, thus contributing to the increased potency (Weglarz-Tomczak et al., 2021). As ebselen has inhibitory activity against numerous targets, it is unlikely to be a viable candidate for further development as a clinical PLpro inhibitor.

## Discussion

The outbreak of COVID-19 has caused a global concern and seriously affects the living styles of most people. PLpro, a multi-functional protease of SARS-CoV-2, can not only digest the polyprotein precursor to generate non-structural proteins but also mitigate the RIG-I-mediated innate immunity triggered by viral infection (Klemm et al., 2020; Shin et al., 2020). Its immunomodulatory effects play an important role in disease progression. Moreover, PLpro is a highly conserved protein among coronaviruses, thus is an ideal target to develop broad-spectrum inhibitors against SARS-CoV, SARS-CoV-2 and future emerged coronavirus strains. This review updates the research progress in developing novel and potent inhibitors of SARS-CoV-2 PLpro, which may reveal some insights into future drug discovery and new strategies for the COVID-19 combat.

Generally, PLpro seems to be a greatly overlooked drug target that lacks research. Numerous research efforts have focused on developing inhibitors of Mpro, another cysteine protease of SARS-CoV-2, but relatively few have focused on PLpro inhibition. Part of the reason can be that PLpro is relatively more challenging to target in the comparison of Mpro. One of the challenges is the presence of homologous host DUBs. Thus, the specificity of the PLpro inhibitor is an important parameter that deserves more attention in the PLpro inhibitor discovery.

Even though a variety of inhibitors against SARS-CoV-2 PLpro have been found as mentioned above, a large portion of hits compounds identified in high-throughput screening and virtual screening have not been further evaluated. For those newly identified inhibitors, *in vitro* studies in a SARS-CoV-2 setting are required to determine its potency. Many studies only evaluated the inhibition of PLpro protease activity by the inhibitor, but ignored the evaluation of its antiviral activities which significantly contribute to the ultimate drug efficacy. As far as the antiviral activity is concerned, most studies conducted this cell-based assay in vero E6 cells which is responsible for the limitation. It is better to perform the antiviral assay in human cells, such as Calu3 cells or normal human airway epithelial cells, the result of which would give different but more clinically relevant EC_50_ values. Moreover, little has been done to test the absorption, distribution, metabolism, and excretion (ADME) and toxicity of these inhibitors. It is necessary to establish a suitable animal model for more rigorous *in vivo* effect evaluation. Then subsequent clinical candidate selection can be possible.

As for mechanistic studies, few structures of inhibitors in complex with PLpro have been resolved. Co-crystallization combined with computational methods can accelerate the disclosure of interaction modes between the inhibitors and PLpro, which can inform future structure-based drug design. Some achievements have been made in the optimization of lead compounds such as GRL0617 and ebselen. Also, additional *in vitro* and *in vivo* evaluations of these potent derivatives are needed in the future.

Drug repurposing is an important strategy in drug discovery. Several existing broad-spectrum antiviral drugs such as dual inhibitors, disulfiram and ebselen, have shown robust inhibition of viral protease and are undergoing clinical trials. These drugs may favour the first line of defence for rapid response. As the high infectivity of SARS-CoV-2 has provoked continuous outbreaks all over the world, the comprehensive applications of drug repurposing, new leads identification, and structure-based leads optimization are expected to further accelerate the discovery process and develop specific inhibitors that are safe, effective and well-tolerated for COVID-19 therapy.

In conclusion, though some encouraging results are available, the drug discovery of SARS-CoV-2 PLpro is still far from meeting the needs. Future researches need to fill the above-mentioned vacancies for developing a promising clinical candidate and an effective PLpro inhibitor with both potency and selectivity eventually.
